# Salvage Therapy With Polatuzumab Vedotin, Bendamustine, and Rituximab Prior to Allogeneic Hematopoietic Transplantation in Patients With Aggressive Lymphomas Relapsing After Therapy With Chimeric Antigen Receptor T-Cells—Report on Two Cases

**DOI:** 10.3389/fonc.2021.737645

**Published:** 2021-09-16

**Authors:** Kristin Gerhardt, Madlen Jentzsch, Thomas Georgi, Aleksandra Sretenović, Michael Cross, Enrica Bach, Astrid Monecke, Sabine Leiblein, Sandra Hoffmann, Milena Todorović, Jelena Bila, Osama Sabri, Sebastian Schwind, Georg-Nikolaus Franke, Uwe Platzbecker, Vladan Vučinić

**Affiliations:** ^1^Leipzig Medical Center, Clinic and Policlinic for Hematology and Cell Therapy, University of Leipzig, Leipzig, Germany; ^2^Leipzig Medical Center, Department of Nuclear Medicine, University of Leipzig, Leipzig, Germany; ^3^Clinical Center of Serbia, Institute for Hematology, University of Belgrade, Belgrade, Serbia; ^4^Leipzig Medical Center, Institute for Histopathology, University of Leipzig, Leipzig, Germany

**Keywords:** DLBCL, polatuzumab vedotin, CAR (chimeric antigen receptor) T cells, allogeneic hematopoeitic stem cell transplantation, PMBCL

## Abstract

Up to 60% of patients with aggressive B-cell lymphoma who receive chimeric antigen receptor (CAR) T-cell therapy experience treatment failure and subsequently have a poor prognosis. Allogeneic hematopoietic stem cell transplantation (alloHSCT) remains a potentially curative approach for patients in this situation. Induction of a deep response prior to alloHSCT is crucial for long-term outcomes, but the optimal bridging strategy following relapse after CAR T-cell therapy has not yet been established. Polatuzumab vedotin, an antibody drug conjugate targeting CD79b, is a novel treatment option for use in combination with rituximab and bendamustine (Pola-BR) in relapsed or refractory disease. Patients: We report two heavily pretreated patients with primary refractory diffuse large B-cell lymphoma (DLBCL) and primary mediastinal B-cell lymphoma (PMBCL) respectively who relapsed after therapy with CAR T-cells with both nodal and extranodal manifestations of the disease. After application of three courses of Pola-BR both patients achieved a complete metabolic remission. Both patients underwent alloHSCT from a human leukocyte antigen (HLA)-mismatched donor following conditioning with busulfan and fludarabine and are disease free 362 days and 195 days after alloHSCT respectively. We conclude that Pola-BR can be an effective bridging therapy before alloHSCT of patients relapsing after CAR T-cell therapy. Further studies will be necessary to define the depth and durability of remission of this salvage regimen before alloHSCT.

## Introduction

Chimeric antigen receptor (CAR) T-cell therapy represents a potentially curative option for primary refractory patients with diffuse large B cell lymphoma (DLBCL) or primary mediastinal B-cell lymphomas (PMBCL), resulting in durable remissions in approximately 40%–50% of cases (1–5). However, the prognosis of patients relapsing after CAR T-cell therapy is dismal and optimal salvage treatment options have not been defined.

Allogeneic hematopoietic stem cell transplantation (alloHSCT) is a potentially curative approach for these patients (6), provided they have a good performance status and a suitable stem-cell donor (7). A deep remission prior to alloHSCT improves the outcome significantly, emphasizing the importance of an optimal bridging strategy to alloHSCT following relapse after CAR T-cell therapy (8).

Polatuzumab vedotin is a novel antibody drug conjugate targeting CD79b and delivering monomethyl auristatin E (MMAE), which is an inhibitor of microtubule formation (9, 10). After binding to CD79b on the B-cell surface, the conjugate is internalized and MMAE released into the cell, which induces apoptosis and inhibits mitosis. Polatuzumab vedotin in combination with Bendamustine and rituximab (Pola-BR) has recently been licensed for treatment of patients with relapsed or refractory DLBCL (11). However, reports on Pola-BR being used as a bridging therapy to alloHSCT in patients who relapse after CAR T-cell therapy have not been published so far.

### Patient 1

A 47-year-old Caucasian male was diagnosed in 2018 with DLBCL in stage IVB according to the Ann-Arbor classification (12, 13) with enlargement of cervical, axillary, and intra-abdominal lymph nodes, bone involvement, and bone marrow infiltration. The immunophenotype of bone marrow showed positivity for CD45, CD19, CD20, CD24, FMC7, CD22, and CD5, weak positivity for CD10, light-chain restriction for κ, partial positivity for CD38, and negativity for CD34, CD23, CD103, CD200, and CD25. Cytogenetic investigation revealed a complex aberrant hyperdiploid clone in 15 of 15 examined metaphases [49,XY,t(3;12)(p21;q24),del(5)(q13q23),der(11)t(1;11)(q32;p15),+16,+18,+15].

The immunohistochemistry of lymph nodes showed TdT-, CD20+, CD3-, bcl2+, bcl6-/+, CD10-, MUM1+, Cyclin D1-, CD23-, CD68-, and Ki67+ (positivity in 80% of cells) and no staining for MYC protein.

Prior to presentation in our center, the patient was progressive under treatment with R-CHOP (rituximab 375 mg/m^2^, cyclophosphamide 750 mg/m^2^, doxorubicin 50 mg/m^2^, vincristine 1.4 mg/m^2^, prednisolone 100 mg), and R-DHAP (rituximab 375 mg/m^2^, dexamethasone 40 mg, cytarabine 2,000 mg/m^2^, cisplatin 100 mg/m^2^). Neither stem cell mobilization with chemotherapy nor steady-state mobilization of stem cells was possible. After the addition of plerixafor to the regimen, a large-volume apheresis was performed. However, the stem-cell yield was insufficient for high-dose therapy, with a total of 0.6 × 10^6^ CD34^+^ cells/kg body weight being collected.

**Figure 1:**
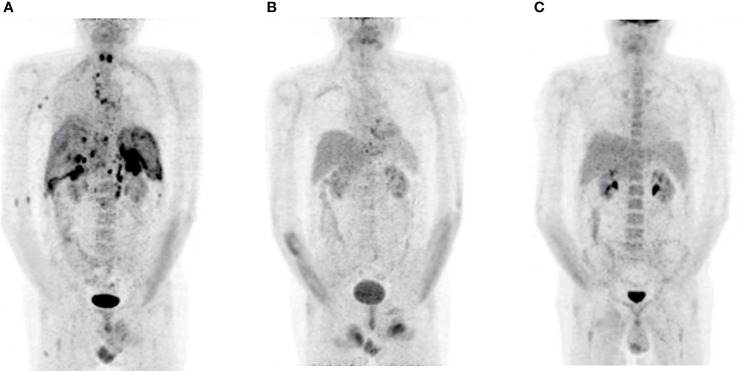
FDG PET imaging patient 1. **(A)** Relapse 6 months after the therapy with CAR T-cells (prior to Pola-BR). **(B)** Complete metabolic remission after three courses of Pola-BR. **(C)** Confirmed complete metabolic remission 12 weeks after alloHSCT.

Although the staging examinations showed bone marrow involvement, 11.2 × 10^9^ autologous T-lymphocytes could be separated for planned therapy with CAR T-cells (tisagenlecleucel). Following bridging therapy with R-Dexa BEAM (rituximab 375 mg/m^2^, dexamethasone 24 mg BCNU 60 mg/m^2^, etoposide 75 mg/m^2^, cytarabine 200 mg/m^2^, melphalan 20 mg/m^2^) (14), a lymphodepleting therapy with fludarabine and cyclophosphamide was followed by reinfusion of tisagenlecleucel. The F-18 fluorodesoxyglucose positron–emission-tomography (FDG PET) computer tomography (CT) prior to lymphodepletion showed non-responding active disease with confirmed bone marrow infiltration. Although the staging 3 months after CAR T-cell therapy showed a complete metabolic remission, the FDG PET CT 6 months after infusion demonstrated a pathological uptake in pleural effusion and lymph node mediastinal, at the liver hilus, and infradiaphragmal paraaortal, as well as in the spleen, and the left adrenal gland (**Figure 1A**). Flow cytometry of pleural effusion confirmed the relapse and positivity for both CD19 and CD79b.

We initiated a salvage therapy with a total of three cycles of Pola-BR (rituximab 375 mg/m^2^, polatuzumab vedotin 1.8 mg/kg, Bendamustine 90 mg/m^2^), resulting in a complete metabolic remission (**Figure 1B**). The immunochemotherapy proceeded without relevant side effects.

After a conditioning regime with busulfan (3.2 mg/kg/day) on days -6 to -4, fludarabine (25 mg/m^2^/day) on days -8 to -4, and cyclophosphamide (60 mg/kg) on days -3 and -2 (15), the patient underwent alloHSCT from an HLA-A antigen-mismatched unrelated male donor. He received a total of 12.3 × 10^6^ CD34+ cells/kg body weight and 2.4 × 10^8^ CD3 cells/kg body weight.

Immunosuppression consisted of tacrolimus, post-transplantation cyclophosphamide on d+3 and d+4, and mycofenolatmofetil (MMF) from d+5, which was tapered according to institutional guidelines. A cytomegalovirus (CMV) reactivation occurred 5 days after alloHSCT and a polyomavirus cystitis 41 days later. Both complications responded completely to conservative therapy. Substitutions of intravenous immunoglobulins were necessary due to a previously diagnosed secondary humoral immune deficiency. The FDG PET CT performed 12 weeks after alloHSCT showed complete metabolic remission (**Figure 1C**).

A mild acute graft *vs*. host disease (GvHD) of the skin (stage I) occurring on day +81 resolved after therapy with local corticosteroids. The complications like veno-occlusive disease (VOD) or sinusoidal obstruction syndrome (SOS) did not occur. The immunosuppression was discontinued 202 days after alloHSCT. The patient is in a persisting complete metabolic remission 362 days after alloHSCT. The immune status showed the normalization of CD3+ count, CD3+ CD4+ cells are light decreased with 434/µl, and the CD19+ cells were detected with normalization of immunoglobulin-G in serum.

### Patient 2

A 48-year-old Caucasian female was diagnosed with a primary mediastinal B cell lymphoma (PMBCL) in stage IIA according to the Ann-Arbor classification with a mediastinal tumor mass and cervical lymph-node involvement. The immunohistochemistry showed positivity for CD23, bcl-2, and FOXP1 and negativity for CD138, CD5, cycline-D1, and CD10 and no staining for MYC protein. The patient was refractory to two therapy lines with R-CHOEP (rituximab 375 mg/m^2^, cyclophosphamide 750 mg/m^2^, doxorubicin 50 mg/m^2^, vincristine 1,4 mg/m^2^, etopiside 100 mg/m^2^, prednisolone 100 mg) and R-DHAP (rituximab 375 mg/m^2^, dexamethasone 40 mg, cytarabine 2,000 mg/m^2^, cisplatin 100 mg/m^2^) prior to therapy with CAR T-cells (axicabtagene ciloleucel). A successful apheresis of lymphocytes with 9.78 × 10^9^ CD3+ cells was performed in November 2019. The reinfusion of axicabtagene ciloleucel was administered on January 14, 2020, without significant complications.

**Figure 2:**
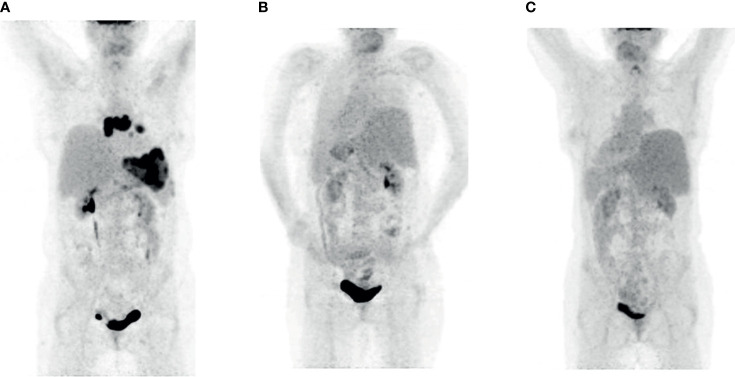
FDG PET imaging patient 2. **(A)** Relapse seven months after the therapy with CAR T-cells (prior to Pola-BR). **(B)** Complete metabolic remission after three courses of Pola-BR. **(C)** Confirmed complete metabolic remission 52 days after alloHSCT.

Although the FDG PET after 3 months showed a complete metabolic remission, the staging examinations 7 months after the application of CAR T-cells displayed a relapse on multiple localizations with increased glucose metabolism (Deauville score 5) in lymph nodes mediastinal, at the left lung hilus and in a bulky lesion in the left upper abdomen (**Figure 2A**). The bone marrow examination showed no infiltration.

Immunochemotherapy with Pola-BR was initiated in September 2020. The FDG PET after three courses showed a complete metabolic remission (**Figure 2B**). After the conditioning with busulfan, fludarabine, and cyclophosphamide as described in the previous patient, the alloHSCT from an unrelated HLA-matched male donor was performed without relevant complications. The FDG PET CT 133 days afterward confirmed the metabolic complete remission (**Figure 2C**). The immunosuppression was discontinued 98 days after alloHSCT. The patient showed no signs of acute GvHD, VOD, or SOS. One hundred ninety-five days after alloHSCT, the CD3+ CD4+ counts are moderately decreased with 344/µl; no CD19+ cells are detectable with severe hypogammaglobulinemia.

## Discussion

Here we present two primary refractory patients with DLBCL and PMBCL who relapsed 6 and 7 months after therapy with CAR T- cells, respectively, and were able to proceed to alloHSCT after a bridging therapy with Pola-BR. Both patients are alive and in complete metabolic remission 362 and 195 days after alloHSCT.

Relapses after therapy with CAR T-cells have a dismal prognosis, and further therapeutic options are limited (3). In the international multicohort retrospective non-Hodgkin lymphoma research study (SCHOLAR-1), the patients after the second therapy line showed event-free survival of only 6.1 months (16). AlloHSCT represents the only curative treatment option, even if the complications of high non-relapse mortality (35% after 1 year) and a high rate of grade 2–4 acute graft-*versus*-host disease (30%) render this option limited to a relatively small number of patients (15).

Since there is no standard of care for such patients, there is an urgent need to establish an adequate salvage strategy prior to alloHSCT. Even though there is an evident requirement for deep remission prior to alloHSCT, intensive salvage treatment options are often limited due to the cumulative toxicities of prior therapies.

This raises the question if a primary refractory patient should undergo the treatment with CAR T-cells at an earlier stage. The clinical trials comparing the safety and efficacy of CAR T-cell treatment with high-dose therapy in the second therapy line are ongoing (NCT02445248, NCT03391466).

Both reported patients were eligible for myeloablative conditioning with fludarabine in combination with busulfan and cyclophosphamide according to the previously published data (15). Although not superior to myeloablative, the reduced-intensity conditioning regimens are an acceptable alternative for older or comorbid patients (17).

Polatuzumab vedotin has previously shown activity as a monotherapy in relapsed and refractory DLBCL (18), but the combination with rituximab resulted in overall response rates of 13%–56%, although the rates of CR were low (13%–56%) (19). In a phase Ib/II study, the Pola-BR combination showed superiority over BR in terms of response in patients either ineligible for or relapsed after high-dose therapy. The patients treated with Pola-BR *vs*. BR showed CR rates of 40% and 17.5% respectively, with longer median progression-free survival (median 9.5 *vs*. 3.7 months) and longer median overall survival (12.4 *vs*. 4.7 months). These data suggest that the combination Pola-RB is an effective salvage therapy, but obviously a further consolidation therapy is necessary. None of the patients included in the trial was previously treated with CAR T-cells. The combination Pola-RB is licensed for use in combination with rituximab and Bendamustine in relapsed or refractory disease (11, 20).

Prior to alloHSCT, both reported patients underwent the therapy with Pola-BR.

In the real-world setting, Pola-BR is frequently used as bridging therapy prior to administration of CAR T-cells (21, 22). However, we are not aware of any other report of remission being induced with Pola-BR in patients who relapsed after therapy with CAR T-cells.

Spiegel et al. recently published the, until now, biggest trial reporting about salvage treatments after CAR T-cells. Interestingly, none of the reported patients was treated with Pola-BR (23).

Pola-BR is not the only promising option for such patients. A further potential salvage option prior to alloHSCT is tafasitamab, a monoclonal Anti-CD19 antibody applied in combination with Revlimid. In a recently published multicenter, prospective, single-arm phase II study (L-MIND), 80 adult refractory and relapsed patients with DLBCL ineligible for auto HSCT with a median of two prior treatment lines were included. Objective responses were seen in 60% of these patients, with a median duration of 21.7 months (24). However, although Tafasitamab-Revlimid has FDA approval, the administration in Europe is currently possible only in terms of compassionate use programs. Furthermore, the application of this therapy is limited to CD19-positive relapses after CAR T-cell therapy.

Another potential agent is loncastuximab tesirine, a humanized anti-CD19 antibody conjugate with pyrrolobenzodiazepine dimer cytotoxin (25) recently approved by FDA (26). In a phase II trial, loncastuximab tesirine as single agent was applied on 145 patients with r/r DLBCL with overall response rates of 48.3% (24.1% complete remissions) with median progression-free survival of 4.9 months. Fourteen patients underwent subsequent treatment with anti-CD19 CAR T-cells after treatment with loncastuximab tesirine. In all patients with biopsies (10 of 14), CD19 expression after treatment with loncastuximab tesirine was found. Thirteen included patients had previous treatment with CAR T-cells with similar overall response rates to the general population, suggesting that loncastuximab tesirine is an adequate strategy both to bridging with CAR T-cells and as a salvage after CAR T-cell therapy.

Limited by only two patients, we assume that Pola-BR could be an effective salvage standard prior to alloHSCT in patients relapsing after CAR T-cell therapy. Both our patients are currently in persistent complete metabolic remission 362 and 195 days after alloHSCT with prior bridging with Pola-RB. Further studies will be necessary to define the optimum number of courses of Pola-RB and long-term outcomes of this treatment regime.

## Data Availability Statement

The raw data supporting the conclusions of this article will be made available by the authors, without undue reservation.

## Ethics Statement

Ethical review and approval was not required for the study on human participants in accordance with the local legislation and institutional requirements. The patients/participants provided their written informed consent to participate in this study. Written informed consent was obtained from the individual(s) for the publication of any potentially identifiable images or data included in this article.

## Author Contributions

KG and VV wrote the manuscript. MJ, MC, UP, SS, and G-NF critically reviewed the manuscript. TG and OS conducted the imaging examinations and provided the pictures. EB, AM, SL, and G-NF performed the laboratory diagnostics. AS, SH, MT, and JB provided administrative support. All authors contributed to the article and approved the submitted version.

## Funding

The authors acknowledge support from the German Research Foundation (DFG) and Universität Leipzig within the program of Open Access Publishing.

## Conflict of Interest

G-NF, UP, and VV received Honoraria from Novartis and Gilead. MJ and SS received Honoraria from Novartis.

The remaining authors declare that the research was conducted in the absence of any commercial or financial relationships that could be construed as a potential conflict of interest.

## Publisher’s Note

All claims expressed in this article are solely those of the authors and do not necessarily represent those of their affiliated organizations, or those of the publisher, the editors and the reviewers. Any product that may be evaluated in this article, or claim that may be made by its manufacturer, is not guaranteed or endorsed by the publisher.
